# A Microfluidic Approach for Quantitative Study of Spatial Heterogeneity in Bacterial Biofilms

**DOI:** 10.1002/smsc.202200047

**Published:** 2022-09-20

**Authors:** Yuzhen Zhang, Yumin Cai, Lingbin Zeng, Peng Liu, Luyan Z. Ma, Jintao Liu

**Affiliations:** ^1^ Center for Infectious Disease Research School of Medicine Tsinghua University Beijing 100084 China; ^2^ Tsinghua-Peking Center for Life Sciences Beijing 100084 China; ^3^ Department of Biomedical Engineering School of Medicine Tsinghua University Beijing 100084 China; ^4^ State Key Laboratory of Microbial Resources Institute of Microbiology Chinese Academy of Sciences Beijing 100101 China

**Keywords:** biofilms, microfluidics, quantitative analysis, spatial heterogeneity

## Abstract

Bacterial biofilms play essential roles in ecological environments and in human health. The spatial heterogeneity of biofilms is crucial to their resistance and collective behavior, while quantitative analysis of these biofilm‐specific features is limited. Here, a microfluidic approach is developed to address this issue. Through a special design of microfluidic chamber and spatially controllable bacteria seeding, biofilms are cultivated with customized semi‐2D structure, which enables quantitative measurements of spatially heterogeneous features with time‐lapse microscopy. The advantages of the proposed method are demonstrated via two examples on biofilm homeostasis and stress response, respectively, where the functionally important spatiotemporal dynamics is delineated. In homeostasis, it is found that *Pseudomonas aeruginosa* biofilms use spatially organized extracellular matrix to preserve iron chelators within their boundaries while maximizing free sharing within the community. In stress response, the spatial distribution of antibiotics in biofilms and how a change in energy metabolism leads to redistribution of drugs over space are elucidated. The proposed method enables cultivating biofilms formed by a wide range of species and even multiple biofilms, which provides a tractable approach to understanding the spatiotemporal features of biofilms formed by environmentally and clinically important bacteria.

## Graphical abstract

Spatial heterogeneity of biofilms is crucial to their resistance and collective behavior, while quantitative analysis of these biofilm‐specific features is limited. A microfluidic approach to address this issue is developed. Through a special design of microfluidic chamber and spatially controllable bacteria seeding, biofilms are cultivated with customized semi‐2D structure, which enables quantitative measurements of spatially heterogeneous features.

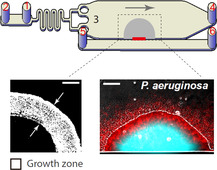

## 1 Introduction

Bacteria usually live in communities, which is beneficial for their survival.^[^ 1, 2, 3 ^]^ Many communities exist on surfaces, where the bacteria secrete extracellular polymeric substances such as proteins, polysaccharides, and extracellular DNA, which enable the bacteria to form dense aggregates of biofilms.^[^ ^4^ ^]^ It was estimated that community living accounted for the majority of the bacteria in nature.^[^ ^5^ ^]^ In addition to being environmentally important, biofilms are also closely related to our health. For example, biofilms are often the cause of infections. Moreover, since biofilms are highly tolerant to antimicrobials and to our immune system, those infections are often persistent and are hard to cure.^[^ ^6^ ^]^

A distinguishing feature of bacterial communities such as biofilms is spatial heterogeneity.^[^ ^7^ ^]^ Due to the dense packing of the bacteria and the derived limitation in substance diffusion, spatial gradients often emerge within a such population and were further reinforced by their own metabolic activities. These spatial gradients gave rise to different microenvironments within the community for specific resident bacteria. As a consequence, the bacteria in different regions of the community often display a broad spectrum of physiological states.^[^ 8, 9, 10, 11, 12, 13 ^]^ Moreover, the cross‐regional interplay is ubiquitous in biofilms, as shown in recent studies that metabolic codependence and coordinated signaling contributed to the fitness of the population.^[^ 14, 15, 16 ^]^

Quantitative studies on spatial heterogeneity can help us to gain a deeper understanding of biofilms and their resistance.^[^ ^17^ ^]^ Most of the biofilm studies so far have used agar plate,^[^ 18, 19 ^]^ microtiter plate,^[^ 20, 21 ^]^ or flow cell^[^ 22, 23 ^]^ (**Table** **1**). Agar plate and microtiter plate are high‐throughput and low cost and have been used to screen for genes involved in biofilm formation and for anti‐biofilm drugs. However, they are closed‐system culturing methods, resulting in the change of growth conditions over time as nutrients are consumed and metabolic wastes accumulate, and the change of growth condition is usually undefined. The flow cell method overcomes this limitation, as the biofilm is cultured in a flowing environment, and the growth condition can be maintained constant by a continuous supply of fresh medium. The three aforementioned methods all generate biofilms with complex morphology, which mimics aspects of natural biofilms to a certain degree, but also impose difficulties in quantitative analysis. Microfluidic‐based approaches are generally suitable for quantitative analysis, and multiple designs have been developed.^[^ 24, 25, 26, 27, 28, 29, 30 ^]^ However, existing designs often have various limitations, including: small population size, missing important emergent population‐level properties that require a minimum population size; random seeding of bacteria, resulting in high variability between experiment; prone to clogging, preventing long‐term observation of biofilms.

**Table 1:** Biofilm community cultivation methods

Method	Features	Advantages	Limitations
Agar plate^[^ 18, 19 ^]^	Air–solid interface; Closed system; No flow.	High throughput; Large population size; Low cost; No need for advanced equipment.	Growth condition changes over time and is undefined; Complex morphology, unsuitable for quantitative analysis;
Microtiter plate^[^ 20, 21 ^]^	Liquid–solid interface; Closed system; No flow.	High throughput; Large population size; Low cost; No need for advanced equipment.	Growth condition changes over time and is undefined; Complex morphology, unsuitable for quantitative analysis;
Flow cell^[^ 22, 23 ^]^	Liquid–solid interface; Open system; Controlled flow; Macro‐scale chamber.	Controlled growth condition; Large population size; Mimics natural biofilm in flowing environments; Long‐term tracking of biofilm dynamics;	Low throughput; Complex morphology, unsuitable for quantitative analysis; High medium consumption, high cost for expensive reagents; Requires confocal microscope.
Existing microfluidic methods^[^ 24, 25, 26, 27, 28, 29, 30 ^]^	Liquid–solid interface; Open system; Controlled flow; Micro‐scale chamber.	Controlled growth condition; Simplified morphology, suitable for quantitative analysis; Low medium consumption, low cost for expensive reagents; Observe with a regular microscope, compatible with single‐cells analysis.	Low throughput; Small population size, missing many collective behaviors and spatial heterogeneity. Random seeding of bacteria, high variability between experiments; Prone for clogging, preventing long‐term culturing; Requires special equipment;
Our microfluidic method	Liquid–solid interface; Open system; Controlled flow; Micro‐scale chamber.	Controlled growth condition; Simplified morphology, suitable for quantitative analysis, while preserving spatial heterogeneity in natural biofilms; Large population size, preserving collective behaviors in natural biofilms; Spatially controlled seeding of bacteria, high reproducibility, suitable for a broad range of bacteria; No clogging, suitable for long‐term tracking of biofilm dynamics; Low medium consumption, low cost for expensive reagents; Observe with a regular microscope; compatible with single‐cells analysis.	Low throughput; Requires special equipment.

Here we developed a new microfluidic design that resolved many of the limitations mentioned earlier. Briefly, this approach is based on a special design of the microfluidic chamber and spatially controlled bacteria seeding, which enabled us to cultivate bacterial biofilms with a customized semi‐2D structure containing millions of cells for an extended period of time (up to 7 days), and maintain precise control over the growth environment throughout the entire duration. To demonstrate the advantages of our approach, we present two examples, which revealed how spatial heterogeneity contributed to the homeostasis and stress response of biofilms. In particular, we elucidated how *Pseudomonas aeruginosa* biofilms adopted spatial organization of extracellular matrix to preserve ion chelator, a critical public good for biofilm homeostasis; We also found that the spatial distribution of energy metabolism played critical roles in biofilm response to antibiotics.

## 2 Results

### 2.1 Description of the Method

**Figure 1:**
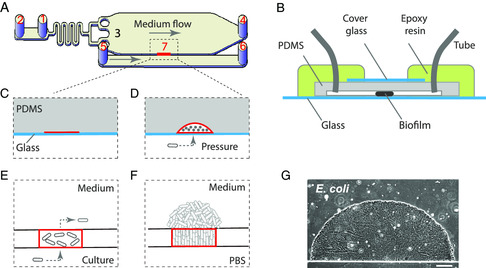
Microfluidic design. A) Schematic diagram of the microfluidic device: 1) medium inlet; 2) medium exchange port; 3) growth chamber; 4) waste outlet; 5,6) bacteria loading channel and ports; 7) bacteria seeding zone (marked in red). B) Side view of the microfluidic chip. C) Side view of seeding zone before loading bacteria. The position (marked in red) is reversibly bonded to the glass, while the other position is irreversibly bonded to the glass. D) Side view of seeding zone during loading bacteria. Planktonic culture of bacteria was injected into the loading channel, because of PDMS being elastic and the seeding zone (marked in red) being reversibly bounding with the glass, the injection pressure created a narrow gap allowing bacteria to pass through. E) Top view of the seeding zone when loading. F) Top view of a biofilm growing out of the seeding zone. G) A phase contrast image of an *Escherichia. coli* biofilm. Scale bar: 100 μm.

**Figure** **1**A–F are schematic diagrams of our microfluidic chip. A key feature is that, different from the random seeding of bacteria to the growth chamber in many microfluidic approaches, we seed the bacteria to a designated location (Figure 1A, marked in red, Experimetal Section) on the side of the growth chamber, which greatly increase the reproducibility of cultured biofilms (more discussions later) and eliminates clogging of the chamber (allowing long‐term experiments). During loading, we inject planktonic bacteria culture into the loading port 5; The injection pressure creates a narrow gap at the seeding zone (Figure 1D), allowing the bacteria to pass through; Some of the bacteria are trapped at the seeding zone, while the rest are flushed out through waste outlet 4 (Figure 1E, Experimetal Section). In this way, we could plant bacteria specifically in the seeding zone. The trapped bacteria later proliferate into the growth chamber, where a fresh medium is continuously supplied. By secreting the extracellular matrix, the bacteria are able to form a stable and densely packed biofilm in the presence of flow (Figure 1E,F).

**Figure 2:**
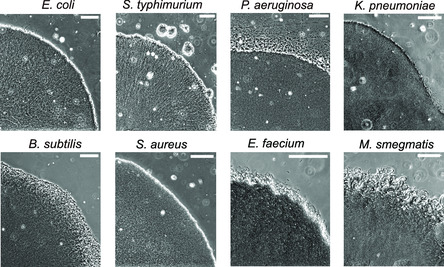
Universality of our design. Phase contrast images of biofilms formed by commonly studied bacterial species. Scale bar: 100 μm.

This design overcomes critical shortcomings of previous approaches to studying biofilms. Many bacteria, especially those of environmental or clinical importance, display a strong preference for adhesion, which often causes unintended seeding of bacteria at random locations and leads to clogging of the microfluidic chip, hindering long‐term tracking of biofilm properties. Here we solved this problem through a specially designed seeding manner. By separating the loading channel from the growth chamber and only seeding bacteria to the designated cell trap, we avoided the main cause of clogging––random adhesion of bacteria in the growth chamber. The controlled seeding also leads to a high reproducibility of biofilms between different experiments, which is essential to quantitative analysis (Figure S1, Supporting Information). Since the seeding mechanism does not rely on the properties of the bacteria, our approach is universal and can be used to culture all the commonly studied bacterial species and potentially many more. Indeed, we successfully cultured biofilms with *Escherichia. coli*, *Salmonella typhimurium*, *P. aeruginosa*, *Klebsiella pneumoniae*, *Bacillus subtilis*, *Staphylococcus aureus*, *Enterococcus faecium,* and *Mycobacterium smegmatis* (**Figure** **2**), which cover gram‐negative, gram‐positive, and mycobacteria. In addition, our design is very flexible and easy to upgrade and adapt (Figure S2A, Supporting Information), and could be extended to study more complex bacterial communities. For example, one could use the upgraded method to study interactions between biofilms of different species (Figure S2B, Supporting Information).

Quantitative analysis had been uncommon in the biofilm research community, especially for the spatial properties of biofilms, and a major obstacle was the morphological complexity of the biofilms in the conventionally used culturing approaches.^[^ 19, 21 ^]^ For example, in the conventionally used flow cell approach, the biofilms usually have irregular 3D structures,^[^ 22, 31 ^]^ which make it difficult to perform quantitative analysis. To study such complicated 3D morphology, confocal microscopy is usually adopted, which gives rise to prolonged scanning time and loss of functionally critical temporal information. Moreover, complicated morphology hindered the quantitative extraction of general principles of spatial heterogeneity from image analysis. By simplifying the morphology of the biofilm, we were able to eliminate this obstacle. We designed a thin growth chamber (6 μm thick). Therefore, the bacteria form a pancake‐like biofilm with uniform thickness (Figure 1F), and conventional microscopes can be used to perform long‐term and high‐frequency imaging. In comparison, the horizontal dimensions of the growth chamber are on the order of millimeters (Figure S3, Supporting Information), allowing the culturing of biofilms for an extended period of time (up to 7 days); in addition, it allows the culturing of biofilms containing millions of cells, which mimics many of the biofilms in nature^[^ ^32^ ^]^ and is essential to the full development of spatial heterogeneity. Finally, together with fluorescent probes, our method can be used to track the spatiotemporal dynamics of molecular activities that are functionally important in biofilms.

We notice that our approach of simplifying 3D biofilms into semi‐2D ones may impact some biofilm properties. For example, the orientation of the bacteria in the biofilm may be affected. Our system certainly mimics biofilms found in confined environments. However, it also captures some properties found in 3D biofilms. We imaged the bacteria in the biofilm with single‐cell resolution (Figure S4, Supporting Information). In the biofilm interior, many but not all bacteria were oriented parallel to the glass substrate, suggesting the biofilm was partially influenced by the boundary effect caused by confinement between the polydimethylsiloxane (PDMS) and the glass substrate (Figure 1B). At the biofilm periphery, most of the bacteria were oriented toward the direction of biofilm expansion; this orientation was likely the result of self‐organization caused by biofilm growth and was also observed in 3D biofilms.^[^ 33, 34, 35 ^]^ In addition to bacteria orientation, another important property captured by our system is spatial heterogeneity, which is one of the most important aspects of biofilm physiology.^[^ ^7^ ^]^ In the following, we discuss spatial heterogeneity in oxygen, nutrient, resource utilization, and antibiotic response.

### 2.2 Spatial Gradient of Oxygen in Biofilm

Oxygen gradient plays important roles in biofilm physiology.^[^ 11, 36 ^]^ In natural biofilms, there are steep O_2_ gradients, and the biofilm interior is usually hypoxic.^[^ 11, 36 ^]^ However, the PDMS material commonly used in microfluidics is gas permeable, which leads to vertical diffusion of O_2_ from the ambient environment into the growth chamber, causing loss of O_2_ gradient. To overcome this problem, we sealed the PDMS with cover glass and epoxy resin (Figure 1B), both of which are gas impermeable; The glass covers the chamber portion of the PDMS, so that the biofilm could be observed with a microscope; The epoxy resin covers the rest of the device, which also provides mechanical support to the tubes. In addition, we made the PDMS layer as thin as possible (1 mm), which minimizes lateral diffusion of O_2_ from the flowing medium through PDMS into the biofilm interior. PDMS has a certain flexibility and becomes more deformable as its thickness is decreased; since the growth chamber has a large lateral dimension but small height, if the thickness of the PDMS layer is further decreased, it will be prone to form permanent bonding with the glass substrate in the middle of the chamber.

**Figure 3:**
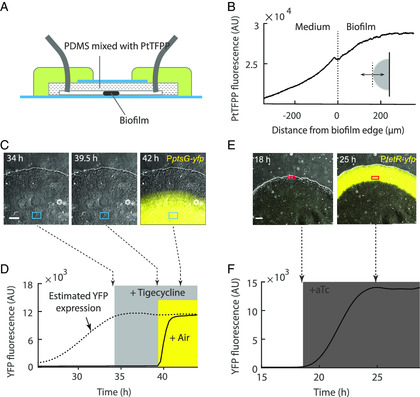
Oxygen gradient in *E. coli* biofilm. A) Oxygen probe PtTFPP imbedded into the PDMS for testing the oxygen distribution in biofilm. B) Spatial profile of PtTFPP fluorescence over an *E. coli* biofilm. The double arrow in the inset illustrates the line along which the profile was measured. C) Time‐lapse images of an *E. coli* biofilm; Composite of phase contrast and YFP (shown in yellow) channels; The YFP channel reveals the activity of the *ptsG* promoter; Scale bar: 100 μm. D) YFP fluorescence intensity at biofilm interior (the region marked by the rectangle in (C). The solid line shows the measured fluorescence value. The dashed line shows the estimated level of YFP protein based on the dashed line in Figure S5C, Supporting Information; The shaded regions represent the durations of tigecycline (2 μg mL^−1^, shown in gray) and air (shown in yellow) treatments, respectively. The dashed arrows indicate the time when the snapshots in (C) were taken. E) Snapshots of an *E. coli* biofilm. Composite of phase contrast and YFP (P*tetR‐yfp* fluorescence, shown in yellow) channels. Scale bar: 100 μm. F) YFP fluorescence at biofilm periphery (the region marked by a rectangle in (E)). The gray shading represents the duration of aTc (anhydrotetracycline, inducer of the *tetR* promoter, 100 ng mL^−1^) treatment. The dashed arrows indicate the time when the snapshots in (E) were taken.

To test the effectiveness of this approach, we embedded the oxygen probe PtTFPP into the PDMS (**Figure** **3**A). The fluorescence of the probe is quenched by O_2_, which can be used as a measure of O_2_ availability.^[^ ^37^ ^]^ Results confirmed that an O_2_ gradient was generated by the biofilm (Figure 3B). To test whether the biofilm interior was hypoxic, we utilized the fluorescent protein YFP, which requires O_2_ to maturate into its fluorescent form^[^ ^38^ ^]^; We constructed a YFP expressing *E. coli* strain driven by the *ptsG* promoter; The promoter is activated by glucose limitation and therefore the YFP would only be expressed at biofilm interior,^[^ ^24^ ^]^ and we should not see YFP fluorescence if the biofilm interior were hypoxic. Indeed, biofilms formed by this strain only showed background fluorescence in our microfluidic chip after growing for 34 h (Figure 3C,D). To confirm that the YFP protein was indeed expressed at the biofilm interior and did not maturate due to lack of oxygen, we cultured biofilms without oxygen limitation (Figure S5A, Supporting Information) and observed YFP fluorescence at the biofilm interior at 34 h (Figure S5B,C, Supporting Information); To further confirm the existence of immature YFP protein in the biofilm shown in Figure 3C, we fed air to the biofilm through the loading channel of the microfluidic chip (Figure 1A, Experimetal Section), and YFP fluorescence immediately emerged (Figure 3C,D). Note, to rule out the possibility that the fluorescence came from newly synthesized YFP during air feeding, we supplemented the protein‐synthesis‐inhibiting antibiotic tigecycline to the biofilm 8 h before feeding air; Figure S5C, Supporting Information, shows that 5 h was more than sufficient to inhibit protein synthesis in the biofilm. Finally, we confirmed that YFP could mature in the periphery region of the biofilm without feeding air (Figure 3E,F). These results showed that our method successfully recapitulated the spontaneously formed oxygen gradient within the biofilm, where the biofilm periphery had access to oxygen, while the biofilm interior was hypoxic.

### 2.3 Nutrient Gradient and Spatially Heterogeneous Growth in Biofilm

**Figure 4:**
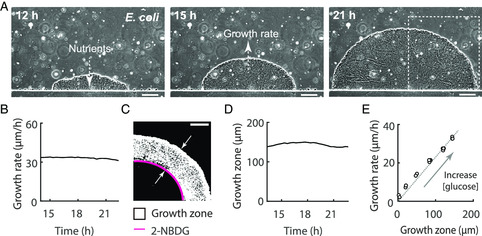
Nutrient gradient and growth of *E. coli* biofilm. A) Phase contrast images of a growing *E. coli* biofilm. Three representative time points are shown. Scale bar: 100 μm. B) The growth rate of the biofilm shown in (A). C) Differencing analysis of the phase contrast images (Methods: Image analysis) revealed a zone of growth in biofilm; 2‐NBDG band (the magenta line) indicates the depth at which the glucose can reach. The dashed rectangular region in (A) is shown. Scale bar: 100 μm. D) The width of the growth zone shown in (C). E) Biofilm growth rate was proportional to the width of the growth zone, and the two were determined by glucose concentration. The glucose concentrations used for the different points are from small to large: 0.22, 2.2, 5.5, 11, 16.5, and 22 mM and the concentration were also shown in Figure S6A, Supporting Information. *n* = 18 biofilms.

Akin to oxygen, nutrient gradients are also formed spontaneously in biofilms. We next quantified these gradients and asked whether these gradients impacted the collective growth of the biofilm. Interestingly, we found the radius of the biofilm increased at a constant rate (**Figure** **4**A,B, Movie S1, Supporting Information, we define it as biofilm growth rate). Image analysis revealed that growth was mainly from the periphery region of the biofilm (Figure 4C, Experimetal Section), and the depth of the growth zone was also constant over time (Figure 4D). Since nutrients penetrate the biofilm through diffusion (Figure 4A), and are consumed by the densely packed bacteria during the process, there is an inward gradient of nutrients in the biofilm. In our experimental setting, the biofilm was cultured with a defined minimal medium with glucose as the carbon source. We suspected that glucose gradient might account for such spatially restricted growth (Figure 4C). To test this, we used a fluorescent glucose analog 2‐NBDG, which is only imported and enriched in the bacteria in during glucose starvation. Hence, its fluorescence is a good indicator of the location of glucose depletion in the biofilm. Using this probe, we observed a glucose depletion band distributed exactly at the boundary between the growth zone and the non‐growing biofilm interior (Figure 4C). Moreover, when we increased/decreased glucose concentration, both the growth rate and the depth of the growth zone increased/decreased accordingly, and the two quantities are proportional to each other (Figure 4E, S6A, Supporting Information), suggesting the growth was limited by the diffusion of glucose into the biofilm. Finally, we confirmed that the biofilm growth rate was independent of the chamber thickness (Figure S6B, Supporting Information), which gives flexibility to the design of the microfluidic chip.

### 2.4 Spatial Division of Labor and Resource Retention in Biofilm

**Figure 5:**
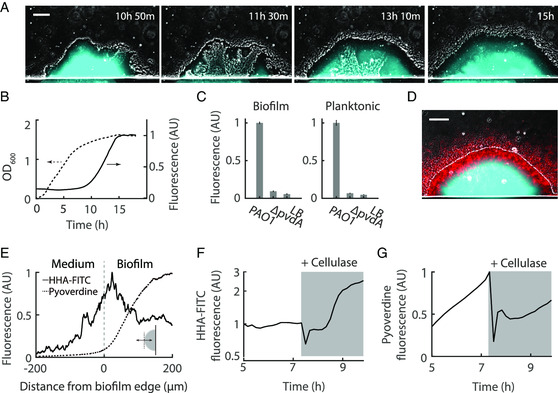
Resource retention by *Pseudomonas aeruginosa* biofilm. A) Time‐lapse images of a *P. aeruginosa* biofilm. Composite of phase contrast and CFP (shown in cyan) channels; Scale bar: 100 μm. B) Optical density and fluorescence intensity (CFP channel) of planktonic bacteria culture. Fluorescence intensity was normalized to the maximum value in the culture. C) Fluorescence intensities of biofilm (interior region) and stationary phase planktonic cultures with or without *pvdA* knockout. Fluorescence intensity in biofilm was normalized to the maximum value in PAO1 biofilm. Fluorescence intensity in planktonic bacteria culture was normalized to the maximum value in PAO1 culture. Error bars represent standard deviation, *n* = 3 biological replicates. D) Staining of biofilm by the fluorescent dye HHA‐FITC (shown in red, 100 ug mL^−1^), which binds specifically to the extracellular matrix component Psl; Scale bar, 100 μm. E) Spatial profile of HHA‐FITC and pyoverdine fluorescence over a *P. aeruginosa* biofilm. The double arrow in the inset illustrates the line along which the profile was measured. Pyoverdine and HHA‐FITC fluorescence were normalized to the maximum value in the biofilm, respectively. F) HHA‐FITC fluorescence at biofilm periphery before and after switching to a medium containing cellulase (84 U mL^−1^, Sigma C2730‐50 mL). G) Pyoverdine fluorescence at biofilm interior.

We next applied our method to biofilms formed by *P. aeruginosa*, which is an important pathogen that causes cystic fibrosis in the lungs.^[^ 39, 40 ^]^ First, we successfully recapitulated the dispersal phenomena well known in *P. aeruginosa* biofilms (**Figure** **5**A),^[^ ^41^ ^]^ showing the compatibility of our method with conventional approaches. In particular, when the biofilm reached the size of ≈240 μm, the interior cells escaped through an opening at the biofilm periphery, leaving behind a hollow shell (Figure 5A, Movie S2, Supporting Information). Concomitant with the dispersal phenomena, we were surprised to find the accumulation of autofluorescence in the interior region of the biofilm under the CFP channel (Figure 5A); Accompanying the dispersion was the simultaneous disappearance of the fluorescence, suggesting loss of the fluorescent substance into the environment. When the biofilm later resealed the opening at its periphery, the fluorescence re‐emerged; Interestingly, we observed fluorescence in the hollow region of the resealed biofilm (Figure 5A, at 13 h 10 m). These results suggested that the fluorescent substance was extracellular and diffusible.

Since the fluorescence was most intense in the biofilm interior, we suspected its accumulation might be related to resource limitation. Using planktonic culture, we confirmed that the autofluorescence emerged when the bacteria entered the stationary phase (Figure 5B). Centrifugation of the stationary phase culture showed that the fluorescence was from the supernatant (Figure S7A, Supporting Information), which confirmed the abovementioned observation that the fluorescent substance in the biofilm was extracellular. Among the substances that *P. aeruginosa* secretes, pyoverdine was known to be fluorescent.^[^ ^42^ ^]^ Spectroscopic analysis showed that the excitation and emission spectrum of the stationary phase supernatant was consistent with that of the pyoverdine (Figure S7B, Supporting Information). Finally, when we knocked out the pyoverdine synthesis gene *pvdA*, the autofluorescence in the biofilm and in the planktonic culture was reduced to the background level (Figure 5C), confirming the identity of the fluorescent substance as pyoverdine.

Pyoverdine is a siderophore, which is secreted by bacteria to chelate irons from the environment. Since the functioning of this molecule requires re‐uptake, uncontrolled loss of pyoverdine into the environment would be energetically inefficient. Therefore, we wondered whether the biofilm was able to confine pyoverdine within its boundary and limit the loss to the flowing medium. Here, we found that the extracellular matrix component Psl secreted by the biofilm showed a spatial pattern complementary to that of pyoverdine and exclusively located at the biofilm periphery (Figure 5D,E, Psl was stained by fluorescently labeled lectin HHA‐FITC^[^ 12, 43, 44 ^]^). Therefore, we speculated it may act as a diffusion barrier to limit the loss of pyoverdine. Consistent with our speculation, when we treated the biofilm with cellulase to hydrolyze Psl,^[^ ^43^ ^]^ both the HHA‐FITC and the pyoverdine fluorescence rapidly dropped to a lower level (Figure 5F,G, Movie S3, Supporting Information); Cellulase did not affect pyoverdine fluorescence in planktonic culture (Figure S8, Supporting Information), ruling out quenching of pyoverdine fluorescence. In contrast, treatment of the biofilm with DNase to degrade the extracellular DNA––another important component of the extracellular matrix, did not have an effect on pyoverdine fluorescence (Figure S9, Supporting Information). These results suggested the preservation of pyoverdine via the spatial organization of extracellular polysaccharides in *P. aeruginosa* biofilm. Such a mechanism uncovered by our method might also account for the preservation of other public goods in biofilms.

### 2.5 Energy Metabolism Determines Spatial Distribution of Antibiotics in Biofilm

Compared with planktonic bacteria, biofilms are more resistant to antibiotics, and biofilm‐related infections require a higher dose of antibiotics and a longer course of treatment.^[^ 6, 45 ^]^ Elucidating the drug resistance mechanisms is important in guiding the clinical therapy for biofilm infections. Hence, we adopted our method (Figure S5A, Supporting Information) to explore the spatial heterogeneity of drug resistance in biofilms.

**Figure 6:**
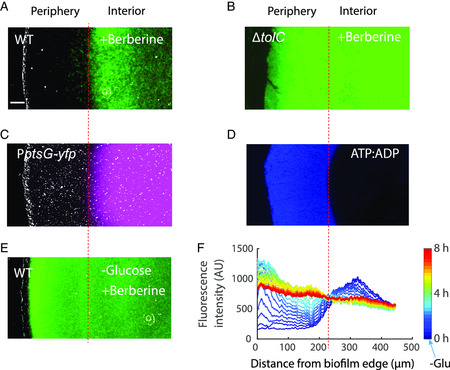
Spatial distribution of berberine in *E. coli* biofilm. A) An image of the spatial distribution of berberine (150 μg mL^−1^) in wild‐type *E. coli* biofilm. Scale bar: 50 μm. B) An image of the spatial distribution of berberine (150 μg mL^−1^) in Δ*tolC* biofilm. C) An image of the spatial distribution of *ptsG* transcription in *E. coli* biofilm. D) An image of the spatial distribution of ATP:ADP in *E. coli* (PercevalHR plasmids) biofilm. E) An image of the spatial distribution of berberine (150 μg mL^−1^) in wild‐type *E. coli* biofilm after removing glucose. F) Spatiotemporal dynamic profiles of distribution of berberine in (E).

We used the antibiotic berberine as an example.^[^ ^46^ ^]^ Berberine is intrinsically fluorescent, which makes it convenient to measure the spatial distribution of the antibiotic. Interestingly, we found berberine was mainly accumulated in the interior region of the *E. coli* biofilm, while the periphery region maintained normal growth (**Figure** **6**A). To understand the mechanism contributing to such spatial distribution, we screened for mutants with deficiencies in common resistance pathways. Among them, we found that *tolC* knockout strain gave rise to a uniform distribution of berberine in the biofilm (Figure 6B); Moreover, it resulted in the rapid killing of the biofilm after adding the same concentration of berberine (Figure S10, Supporting Information). These results showed that the efflux activity, especially that of the *tolC* efflux pump, played an important role in the spatial distribution of berberine in *E. coli* biofilm.

The efflux activity is determined by both the expression level of the efflux pump and the metabolic rate of the bacteria.^[^ 47, 48 ^]^ Since the promoter activity of *tolC* was uniform throughout the biofilm (Figure S11, Supporting Information), we hypothesized that the spatial distribution of metabolic rate in the biofilm resulted in the observed spatial distribution of berberine. Indeed, we found that the region of berberine accumulation in biofilm interior overlapped with that of glucose starvation (Figure 6C). Moreover, the ATP:ADP ratio dropped dramatically in space once glucose is depleted within the biofilm (Figure 6D). Finally, when we removed glucose and blocked the efflux activities, we observed a sharp increase of berberine fluorescence at the biofilm periphery, while drug accumulation remained constant at the biofilm interior (Figure 6E,F, Movie S4, Supporting Information). These results suggest that it is the metabolic rate that determined the overall efflux activities for berberine across space and results in its spatially heterogeneous accumulation in biofilm. Our method uncovered the role of spatial heterogeneity in biofilm response to antibiotics.

## 3 Conclusion

We have developed a universal microfluidic method for the quantitative study of the complex properties of biofilm communities. By limiting the height of the growth chamber, we cultured biofilms with a pancake‐like structure with uniform thickness. Together with time‐lapse microscopy and fluorescent probes, it enabled quantitative measurements of the spatial heterogeneity and spatiotemporal dynamics of biofilms. We developed a new seeding strategy. This strategy has no restriction on the types of bacteria, and the location and number of inoculation sites can be flexibly designed, which expands the application scenarios of the chip. Moreover, we used cover glass and epoxy resin sealing to block air penetration through the PDMS. This design leads to the natural oxygen gradient in the biofilm and thus faithfully reproduces the physiological environment in native settings.

Based on the method developed here, we uncovered diffusion‐limited growth in biofilm (Figure 4), public goods retention via the spatial deployment of diffusion barrier (Figure 5), and spatial heterogeneity of biofilm response to antibiotics (Figure 6). It revealed the rich heterogeneities and derived collective behaviors in spatially structured biofilms, which has the potential to delineate more governing principles in the homeostasis and stress response of biofilms. We are integrating this method into the development of a spatial multi‐omics approach for biofilms research, which will catalyze efforts toward a quantitative and systematic understanding of biofilm communities.

## 4 Experimental Section

4.1

4.1.1

##### Microfluidic Chip Fabrication

The microfluidic chip was designed and manufactured in‐house. A scaled drawing of the microfluidic design is shown in Figure S3, Supporting Information. The master mold of the chip was fabricated using the Maskless Mold Fabrication Method from BlackHole Lab (France), and performing lithography on glass slides using a photoresist from Microchemicals (AZ4562). The microfluidic chips were made with PDMS and glass slides. The PDMS part was made by pouring a 10:1 v v^−1^ mixture of Sylgard 184 elastomer and curing agent (Dow Corning, USA) on the master mold, and curing the mixture for 2 h in an oven at 80 °C. The cured PDMS was carefully peeled off from the mold and punched with holes at the inlet and outlet ports. Then we pasted a narrow (0.5–1 mm) strip of 3M Scotch Magic Tape to a designated location of the PDMS (on the barrier between the growth chamber and the loading channel, and perpendicular to the barrier), and treated the PDMS with plasma for 2 mins (SoftLithoBox, BlackHole Lab, France). After the plasma treatment, the tape was quickly removed, and the PDMS was immediately bounded with a glass slide or cover glass (depending on the magnification of microscopic observations later on). The tape prevented the plasma from reaching the covered spot, therefore, creating an unbounded region between the growth chamber and the loading channel, which could be used for bacteria seeding. Finally, the top surface of the PDMS (middle portion) was bounded with cover glass, the inlet and outlet ports were plugged with syringe needles (20 G, 0.91 mm OD × 0.61 mm ID) and PTFE Tubing (1/16″ OD × 1/32 ID), and the rest of the PDMS with epoxy resin (Figure 1B) was sealed.

To visualize the spatial gradient of oxygen, we embedded the oxygen sensor PtTFPP (Frontier Scientific Inc., USA) in PDMS (Figure 3A): The PtTFPP was dissolved in toluene and thoroughly mixed with PDMS before it was cured; Then the mixture was poured on the mold, and toluene was allowed to evaporate while the PDMS was cured. The final PtTFPP concentration was 1 mg mL^−1^.

##### Bacterial Strains

The bacterial strains used in this study were *E. coli* BW25113, *S. typhimurium* ATCC 14 028, *B. subtilis* NCIB 3610, *P. aeruginosa* PAO1 (Δ*paaP*), *K. pneumoniae* ATCC BAA‐1144, *S. aureus* RN4220, *E. faecium* ATCC 35 667, and *M. smegmatis mc* ^2^‐155 (Table S1, Supporting Information).

*E. coli Strains*: Promoters of *E. coli* strains (P*ptsG* and P*tolC*) were amplified from wild‐type *E. coli* BW25113 genomic DNA; The P*tetR* sequence was synthesized by Genewiz (China). The promoter sequences were amplified by PCR and were then introduced to a plasmid containing fluorescent protein coding sequence to generate promoter activity reporters. Plasmids were constructed using the Gibson assembly method and transformed into DH5*α* competent cells (Weidi, China). DNA purification and isolation of plasmids were performed using reagents from Omega Bio‐Tek (USA). PCR reactions were carried out using Q5 High‐Fidelity DNA polymerase (NEB, UK).

*P. aeruginosa* strains: Most of the experiments were based on the PAO1 Δ*paaP* stain, which was less prone to dispersion than wild‐type PAO1 and more suitable for the current study; It was verified that *paaP* knockout does not affect the production of the fluorescent substance (data not shown). The *pvdA* deletion strain were constructed by an unmarked, non‐polar deletion strategy as previously described.^[^ ^49^ ^]^

##### Bacteria Loading and Biofilm Culturing

On the day before the experiment, bacteria from −80 °C glycerol stock were streaked on LB agar plates and incubated at 37 °C overnight. On the next day, a single colony was picked from the plate and inoculated to 5 mL of LB broth in a 50 mL conical tube, and incubated at 37 °C in a shaker. After 12 h of incubation, the culture was centrifuged at a relative centrifugal force of 7000 g for 3 min, and then the pellet was re‐suspended in 0.5 mL biofilm medium and used for loading into microfluidics. The biofilm medium for *E. coli*, *B. subtilis*, *S. typhimurium*, *K. Pneumoniae*, *E. faecium* and *M. smegmatis* was M63B1 (100 mM KH_2_PO_4_, 15 mM (NH_4_)_2_SO_4_, 0.8 mM MgSO_4_, 3 μM vitamin B1, 22 mM glucose, adjusted to pH 7.4 with KOH)^[^ ^50^ ^]^; The medium for *P. aeruginosa* was LB without sodium chloride (LBNS)^[^ ^43^ ^]^; The medium for *S. aureus* was TSB.

The microfluidic chip was controlled using the Elveflow OB1 Mk3 Pressure Controller (ELVESYS, France), which drives medium flow with pressurized air. At first, the medium was pumped into the growth chamber from ports 1 and 5 (Figure 1A) simultaneously, with ports 2 and 6 closed. The pressure was maintained at 15 psi until all the air bubbles disappeared, which usually took 5–10 mins. Then port 5 was switched to planktonic bacteria culture with a pressure of 10–20 psi, while port 1 was maintained at 15 psi; Once bacteria started to enter the growth chamber, the pressures to ports 1 and 5 were adjusted to 1–5 and 1–2 psi, respectively. After ≈1000 bacteria cells were trapped at the seeding zone, we turned off the pressure to port 5, and adjusted the pressure to port 1 to 3 psi. Finally, port 6 was opened and the loading channel was washed by injecting PBS to port 5 for 30 mins (3 psi). During the rest of the experiment, ports 2, 5, and 6 were kept closed, and port 1 was fed with a medium under a constant pressure of 3 psi, and the temperature was kept at 37 °C. To perform perturbations (e.g., adding antibiotics), port 1 was switched to the corresponding new medium, and port 2 was briefly opened until the residues from the previous medium was completely flushed out and the new medium reached port 1 (this operation allowed fast switching of medium). Normally, the biofilm acquires oxygen from the flowing medium; To supply additional oxygen to the interior region of the biofilm (Figure 3C,D), air was pumped into port 5, with port 6 kept open, and the air could then diffuse into the biofilm from the loading channel; To stop the additional oxygen supply, the loading channel was refilled with PBS and ports 5 and 6 were closed.

##### Time‐Lapse Microscopy

The biofilms were observed with phase contrast and fluorescence microscopy. The microscope used was Olympus IX83 (Japan) with Andor's Zyla 4.2 sCMOS camera (UK). To image the entire biofilm, a 10X objective lens was used in most of the experiments. Images were taken every 3 s–20 min, depending on the question.

##### Image Analysis

ImageJ (National Institutes of Health, USA) and MATLAB (MathWorks, USA) were used for data analysis. To detect the region of expansion in a biofilm, image differencing was performed on snapshots of the biofilm from time‐lapse microscopic images. Specifically, the difference between two consecutive phase contrast images was calculated by calculating the absolute difference between corresponding pixels in the two images. Since expansion leads to pixel level changes and no expansion leads to little change, image differencing could reveal the region of expansion in the biofilm.

##### Planktonic Culture Experiments

The culturing and measurement of planktonic *P. aeruginosa* (Figures 5B,C, S7, and S8, Supporting Information) were performed using the SPARK microplate reader (TECAN, Swiss). The bacteria were grown overnight in the LBNS medium in a 37 °C shaker. On the next day, 4 μL of the culture were inoculated to 200 μL of fresh LBNS and incubated in 96 well plates. Optical density (600 nm) and fluorescence intensity (excitation 430 nm, emission 480 nm) were recorded every 10 min. The excitation and emission spectrum of the supernatant (Figure S7B, Supporting Information) were also measured using the plate reader.

##### Statistical Analysis

Fluorescence intensity in the planktonic culture and biofilms of *P. aeruginosa* were normalized, respectively, to the maximum value in each test (Figures 5, S7–S9, Supporting Information). Data were presented as mean ± standard deviation (SD). The error bars represent the SD (Figures 5, S1, S7, and S8, Supporting Information). Sample size (*n*) was no less than 3 biological replicates in every assay (Figures 4, 5, and S1, Supporting Information).

## Conflict of Interest

Y.Z. and J.L. are inventors of a patent related to this work.

## Author Contributions

Y.Z. and J.L. designed the study; Y.Z. and J.L. designed the microfluidic chip; Y.Z., L.Z., Y.C., and Z.C. performed the experiments; Y.Z., L.Z., Y.C., and J.L. analyzed the data; Y.Z. and J.L. wrote the manuscript, all authors discussed the manuscript.

## Supporting information

Supplementary Material

## Data Availability

The data that support the findings of this study are available on request from the corresponding author. The data are not publicly available due to privacy or ethical restrictions.
