# Supplementary material for: A Microfluidic Approach for Quantitative Study of Spatial Heterogeneity in Bacterial Biofilms
Source: Small Sci. 2022 Sep 20;2(10):2200047. doi: 10.1002/smsc.202200047 (PMC11936032; doi:10.1002/smsc.202200047)
Supplement: Supplementary file 1 — Supplementary Material [file SMSC-2-2200047-s001.zip › smsc202200047-sup-0001-SuppData-S1/smsc202200047-sup-0001-SuppData-S1.pdf]

Supporting Information for

A Microfluidic Approach for Quantitative Study of Spatial Heterogeneity in Bacterial Biofilms

Yuzhen Zhang, Yumin Cai, Lingbin Zeng, Peng Liu, Luyan Z. Ma, Jintao Liu*

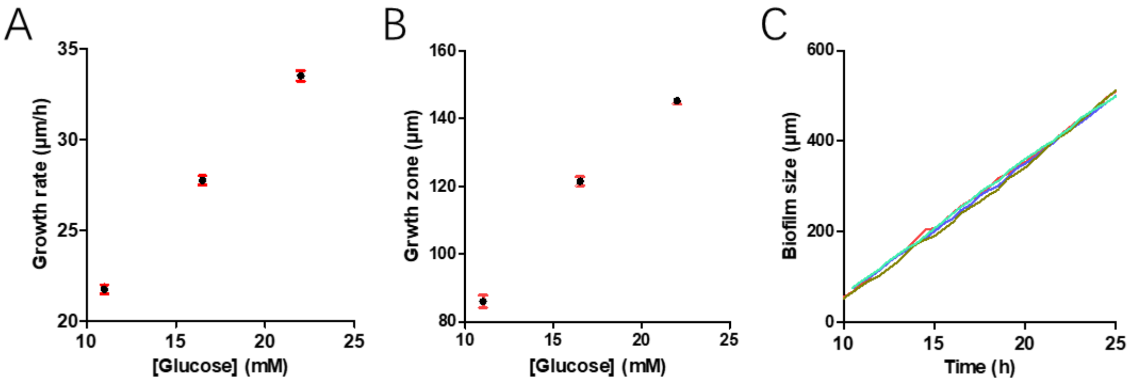

Fig.S1. Repeatability of the growth of the biofilms. (**A**) Growth rate under 11 mM glucose (*n* = 4 biofilms, mean = 21.75 μm/h, s.d. = 0.5 μm/h), 16.5 mM glucose (*n* = 4 biofilms, mean = 27.75 μm/h, s.d. = 0.5 μm/h) and 22 mM glucose (*n* = 4 biofilms, mean = 33.5 μm/h, s.d. = 0.57 μm/h). (B) Growth zone under 11 mM glucose (*n* = 4 biofilms, mean = 86 μm, s.d. = 1.82 μm), 16.5 mM glucose (*n* = 4 biofilms, mean = 121.5 μm, s.d. = 1.29 μm) and 22 mM glucose (*n* = 4 biofilms, mean = 145.2 μm, s.d. = 1 μm). (**C**) Biofilm size increased with time (under 22 mM glucose, *n* = 4 biofilms).

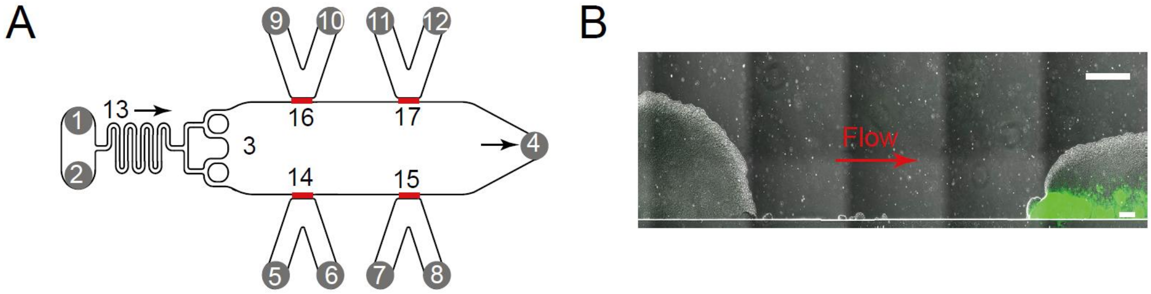

Fig.S2. Flexibility of our design. (**A**) Schematic diagram of our flexible design: 1) medium inlet; 2) medium exchange port; 3) growth chamber; 4) waste outlet; 5 & 6, 7&8, 9&10 and 11&12) bacteria loading channel and ports; 14, 15,16 and 17) bacteria seeding zone (marked in red). (**B**) Snapshot of two biofilms formed by *E. coli* and *P. aeruginosa*; Composite of phase contrast and GFP (indicates *P. aeruginosa*, shown in green) channels; *E. coli* and *P. aeruginosa* were loaded to the seeding zones 1 and 2 respectively, and cultured with the M63B1 medium; Scale bar, 300 μm.

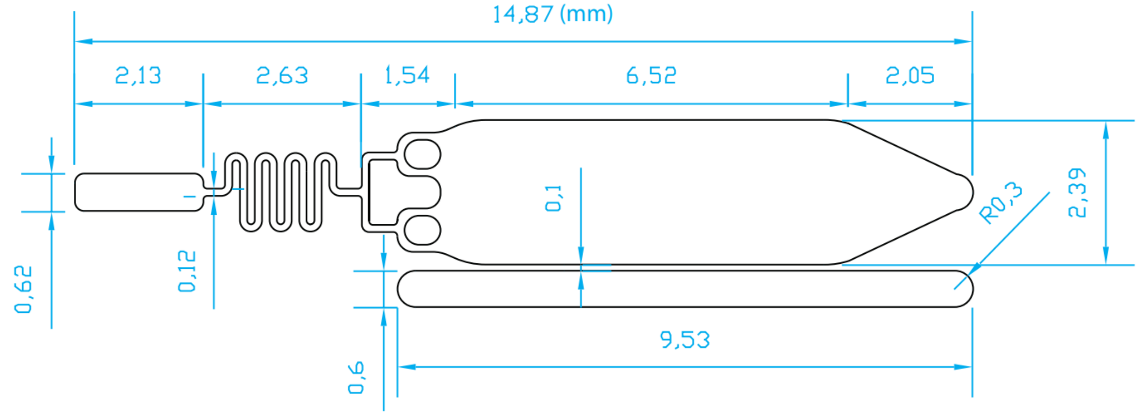

Fig. S3. Dimensions of the microfluidic chip introduced in Fig. 1A.

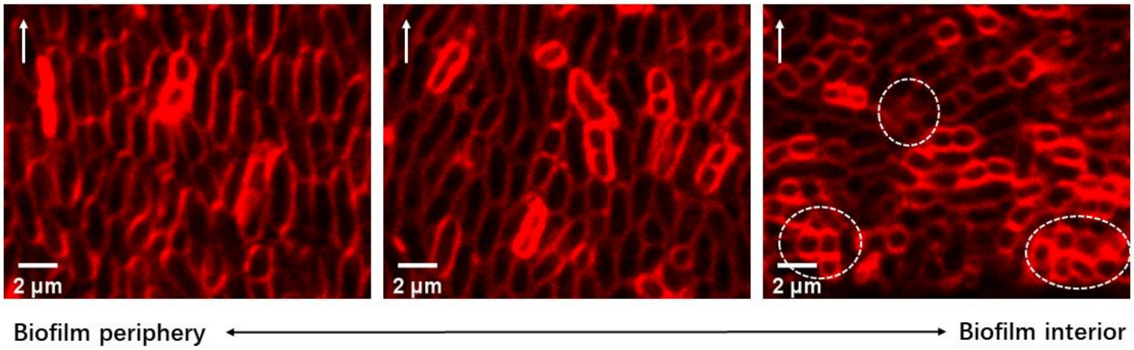

Fig.S4. Bacteria arrangement in a biofilm. 0.5 μg/ml FM4-64 fluorescent dye was used to label the cell membrane. At biofilm periphery, most of the bacteria were oriented toward the direction of biofilm expansion (the white arrow direction), while the internal bacteria are disorderly arranged, and even some bacteria (marked in circle) are perpendicular to the substrate.

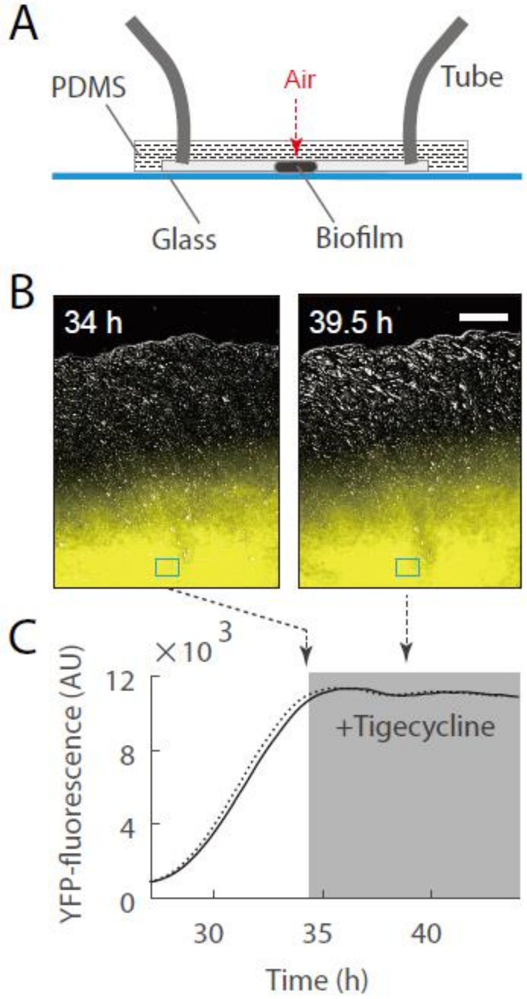

Fig. S5. Biofilm without oxygen limitation. (**A**) Side view of the microfluidic chip; The red arrow indicates how oxygen from the ambient air could diffuse through PDMS to biofilm interior. (**B**) Snapshots of an *E. coli* biofilm; Composite of phase contrast and YFP (P*ptsG-yfp* fluorescence, shown in yellow) channels; Scale bar, 100 µm. (**C**) YFP fluorescence intensity at biofilm interior (the region marked by rectangle in (**B**); The solid line shows the measured fluorescence value; The dashed line shows the estimated level of YFP protein inferred from the solid line, based on the fact that the maturation time of EYFP is 9 mins; The shaded region represents the duration of tigecycline (2 µg/ml) treatments; The dashed arrows indicate the time when the snapshots in (**B**) were taken.

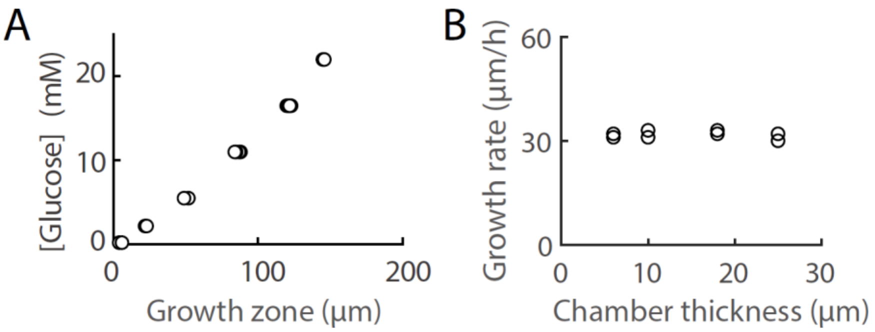

Fig. S6. Growth of *E. coli* biofilm. (**A**) The depth of the growth zone increased with glucose concentration (*n*=18 biofilms). (**B**) Biofilm growth rate was independent of chamber thickness (*n*=8 biofilms).

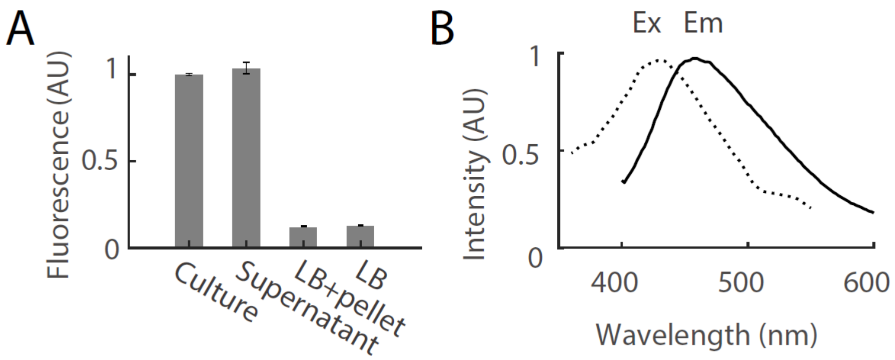

Fig. S7. (**A**) Fluorescence intensities of stationary phase planktonic bacteria culture, its supernatant after centrifugation, its resuspended pellet after centrifugation, and the resuspension medium LB; Error bars represent standard deviation, n = 3 biological replicates. Fluorescence intensities were normalized to the intensity in supernatant. **(B)** Excitation and emission spectrum of stationary phase *P. aeruginosa* culture supernatant.

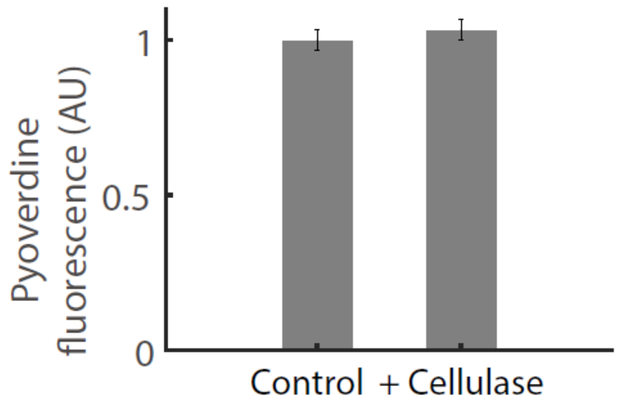

Fig. S8. Effect of cellulase addition (84 U/ml) on pyoverdine fluorescence in stationary phase *P. aeruginosa* culture. The error bars represent standard deviation, n = 3 biological replicates. Fluorescence intensities were normalized to the intensity in control group.

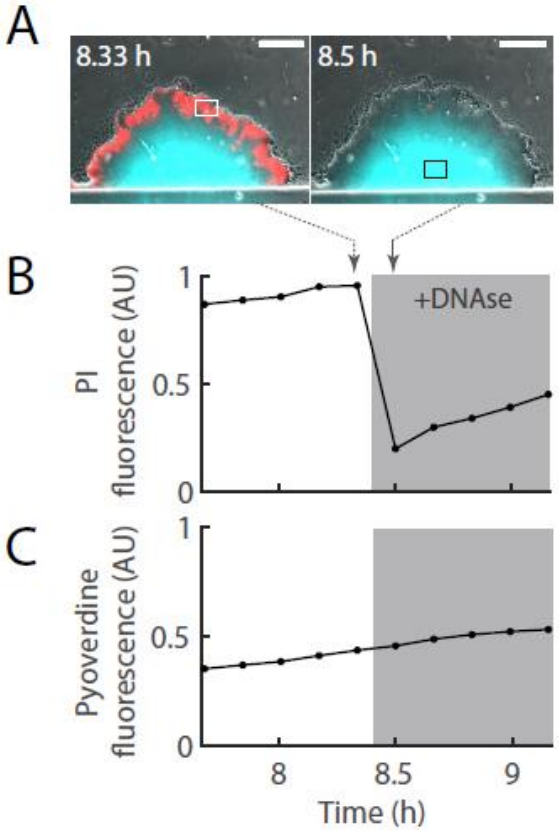

Fig. S9. Treatment of *P. aeruginosa* biofilm with DNase. (**A**) Snapshots of the biofilm; Composite of phase contrast, CFP (pyoverdine fluorescence, shown in cyan), and RFP (PI – propidium Iodide fluorescence, shown in red) channels; Scale bar, 100 µm. (**B**) PI fluorescence at biofilm periphery as indicated by the white rectangle in (**A**); The gray shading indicates duration of DNase treatment (750 U/ml); The dashed arrows indicate the time when the snapshots in (A) were taken. (**C**) Pyoverdine fluorescence at biofilm interior as indicated by the black rectangle in (**A**). PI and Pyoverdine fluorescence were normalized to the maximum value in the biofilm, respectively.

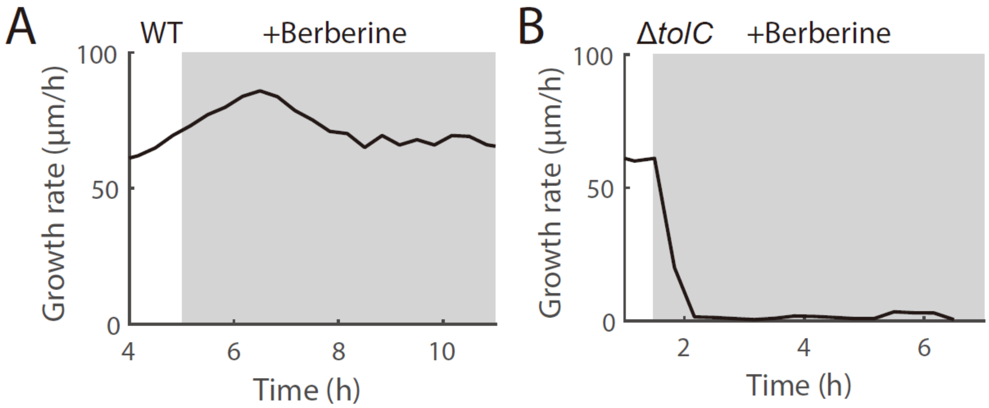

Fig. S10. Biofilm growth rate before and after switching to medium containing 150 μ g/ml berberine. (**A**) WT *E. coli*. (**B**) Δ*tolC*.

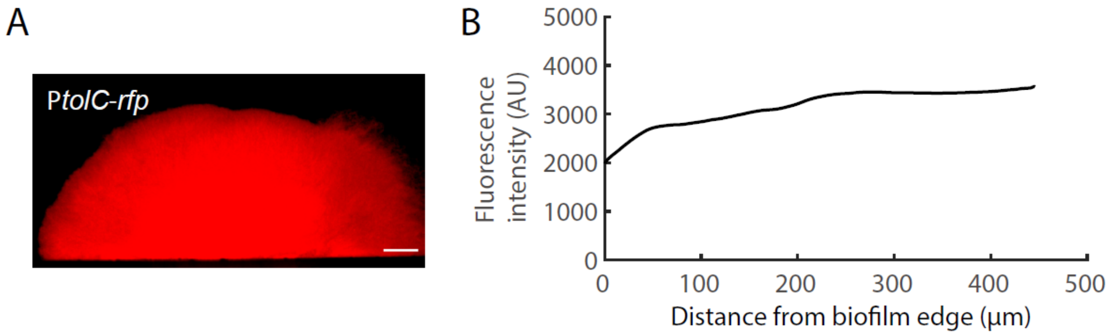

Fig. S11. Distribution of the promoter activity of *tolC* in *E. coli* biofilm. (**A**) A snapshot of *tolC* transcription distribution in biofilm. Scale bar, 100 μm. (**B**). Profile of *tolC* transcription distribution in biofilm.

Table S1. Bacterial Strains used in this study

**Strain**	**Description**
E. coli	BW25113
E. coli with plasmid, pDL30 P*ptsG-yfp*	P*ptsG* was activated by glucose limited
E. coli with plasmid, p15A P*katG-rfp*	P*katG* was activated by H_2O_2
E. coli with plasmid, pSB1C3 P*tetR-yfp*	P*tetR* was induced by aTc
E. coli with knock out of *tolC*	Deficient in efflux pump
E. coli with plasmid, p15A P*tolC-rfp*	*tolC* transcription was represented by YFP fluorescence intensity
E. coli with plasmid, PercevalHR	ATP:ADP sensor
Pseudomonas aeruginosa	PAO1
Pseudomonas aeruginosa Δ*paaP*	Less prone to dispersion in our experiments
Pseudomonas aeruginosa Δ*paaP* Δ*pvdA*	Deficient in pyoverdine synthesis
Salmonella typhimurium	ATCC 14028
Bacillus subtilis	NCIB 3610
Klebsiella pneumoniae	ATCC BAA-1144
7Staphylococcus aureus	RN4220
Enterococcus faecium	ATCC 35667
Mycobacterium smegmatis	mc^2-155

Movie S1. Time-lapse video of an *E. coli* biofilm.

Movie S2. Time-lapse video of a *P. aeruginosa* biofilm. Composite of phase contrast and CFP (shown in cyan) channels.

Movie S3. Time-lapse video of a *P. aeruginosa* biofilm. Composite of phase contrast, CFP (pyoverdine fluorescence, shown in cyan), and GFP (HHA-FITC fluorescence, shown in red) channels; Switched to medium containing cellulose (84 U/ml) after 7h 20m.

Movie S4. Time-lapse video of a *E. coli* biofilm under 150 µg/ml berberine. Composite of phase contrast and YFP (shown in green) channels. Switched to medium with no glucose after 28h.
